# Development of a Two-Component Nanoparticle Vaccine Displaying an HIV-1 Envelope Glycoprotein that Elicits Tier 2 Neutralising Antibodies

**DOI:** 10.3390/vaccines12091063

**Published:** 2024-09-18

**Authors:** Kegomoditswe Malebo, Jeremy Woodward, Phindile Ximba, Qiniso Mkhize, Sanele Cingo, Thandeka Moyo-Gwete, Penny L. Moore, Anna-Lise Williamson, Rosamund Chapman

**Affiliations:** 1 Division of Medical Virology, Department of Pathology, Faculty of Health Sciences, University of Cape Town, Cape Town 7925, South Africa; 2 Institute of Infectious Disease and Molecular Medicine, Faculty of Health Sciences, University of Cape Town, Cape Town 7925, South Africa; 3 Electron Microscope Unit, University of Cape Town, Cape Town 7925, South Africa; 4 MRC Antibody Immunity Research Unit, School of Pathology, University of the Witwatersrand, Johannesburg 2000, South Africa; 5 National Institute for Communicable Diseases of the National Health Laboratory Services, Johannesburg 2192, South Africa; 6 Centre for the AIDS Programme of Research in South Africa, Durban 4001, South Africa

**Keywords:** nanoparticle, mi3, CAP255, DNA, tier 2 neutralising antibodies, human immunodeficiency virus, prime-boost, vaccine

## Abstract

Despite treatment and other interventions, an effective prophylactic HIV vaccine is still an essential goal in the control of HIV. Inducing robust and long-lasting antibody responses is one of the main targets of an HIV vaccine. The delivery of HIV envelope glycoproteins (Env) using nanoparticle (NP) platforms has been shown to elicit better immunogenicity than soluble HIV Env. In this paper, we describe the development of a nanoparticle-based vaccine decorated with HIV Env using the SpyCatcher/SpyTag system. The Env utilised in this study, CAP255, was derived from a transmitted founder virus isolated from a patient who developed broadly neutralising antibodies. Negative stain and cryo-electron microscopy analyses confirmed the assembly and stability of the mi3 into uniform icosahedral NPs surrounded by regularly spaced CAP255 gp140 Env trimers. A three-dimensional reconstruction of CAP255 gp140 SpyTag–SpyCatcher mi3 clearly showed Env trimers projecting from the centre of each of the pentagonal dodecahedral faces of the NP. To our knowledge, this is the first study to report the formation of SpyCatcher pentamers on the dodecahedral faces of mi3 NPs. To investigate the immunogenicity, rabbits were primed with two doses of DNA vaccines expressing the CAP255 gp150 and a mosaic subtype C Gag and boosted with three doses of the NP-developed autologous Tier 2 CAP255 neutralising antibodies (Nabs) and low levels of heterologous CAP256SU NAbs.

## 1. Introduction

Progress is being made toward developing a human immunodeficiency virus (HIV-1) vaccine that effectively induces broadly neutralising antibodies (bNAbs) against HIV-1 envelope glycoproteins (Env) to block viral entry, although such a vaccine is not yet available [1]. This is mainly because HIV-1 Env is poorly immunogenic due to a number of factors, including the low spike density of the glycoprotein on the virion [2,3], a glycan shield derived from the host, limited accessibility of vulnerable epitopes and the presence of incorrectly folded glycoprotein species that misdirect the immune response towards non-neutralising epitopes. As a result, induction of potent bNAb by vaccination remains elusive [4,5,6]. Recently, nanoparticle-based formulations have proved to be a promising approach to enhance immunogenicity, as they provide an optimal biomimetic platform for displaying HIV-1 Env trimers in a conformation that closely resembles that of the native infecting virus [7]. Displaying stabilised HIV-1 Env trimers on nanoparticles (NPs) has been found to improve overall Env immunogenicity. For this reason, a wide range of NPs, including virus-like particles (VLPs), polymers, liposomes/micelles, inorganic NPs and in vivo/in vitro self-assembling NPs, have been investigated to leverage their beneficial properties to elicit protective anti-HIV-1 responses [8]. NPs have a particulate size of ~10–100 nm, which facilitates their unrestricted entry into the lymphatic system and direct drainage to lymph nodes [9], while the dense, repetitive epitope presentation facilitates the cross-linking of B-cell receptors, leading to strong B-cell stimulation and more durable antibody responses [10,11]. In addition, the multimeric nature of NPs provides both dual antigen and adjuvant-like properties, enabling the induction of both humoral and cellular immune responses, which is critical for antiviral protection. Immunization with the Env trimer displayed on the surface of NPs or VLPs also conceals the basal epitopes, minimizing off-target base responses that potentially divert the immune response and impede bNAb formation [4,12].

The computationally designed i301 nanocage is a promising NP synthetic model with increased particle uniformity and thermostability. It is derived from the 2-keto-3-deoxy-phosphogluconate (KDPG) aldolase enzyme of the Entner–Doudoroff pathway of a hyperthermophilic bacterium called *Thermotoga maritima* [13]. i301 is composed of 60 monomeric subunits that assemble together into a dodecahedron structure with 12 pentagonal faces [14]. Homogenously decorated i301 NPs can be expressed in *Escherichia coli* with monomeric subunits fused to either or both termini without causing particle distortion [14]. In HIV-1 vaccine development, He et al. [15] reported the development of modest autologous Tier 2 neutralising antibody responses when rabbits or mice were vaccinated with NPs presenting full-length gp140 trimers with a linker or T cell epitope fused to the C-terminus, joining it to the N-terminus of the i301 nanoparticle [15]. Mutating the C76A and C100A regions of i301 to alter disulphide bond formation resulted in an optimised mi3 NP with increased particle uniformity and thermostability [13]. This is an advantage for vaccine supply in areas where cold-chain storage is hard to maintain [16]. This self-assembling NP has the capacity to display up to 20 trimers [13]. mi3 NPs have been used in vaccine development against several pathogens, including SARS-CoV-2 [17,18,19,20,21], malaria [13] and influenza [22].

However, chemically conjugating or genetically fusing antigens to NPs or VLP subunits can cause misfolding or aggregation [23,24]. Two-component nanoparticle systems, the SpyTag/SpyCatcher system, enable the expression and purification of native antigens of high quality, such as HIV-1 Env trimers, prior to coupling to the nanoparticle for immunisation [25]. SpyCatcher is a 12 kDa protein engineered to form a spontaneous amide bond with its peptide partner SpyTag [13].

Here, we present HIV-1 Env trimers in a high-density arrangement on the surface of a self-assembling mi3 NP platform using the SpyCatcher/SpyTag system. The design, assembly, in vitro characterisation and immunogenicity of these NPs is described in this paper.

## 2. Materials and Methods

### 2.1. Plasmids, Antibodies, Cell Lines, Media and Reagents

The pET28a-MBP SpyTag (Addgene ID: 35050) and pET28a-SpyCatcher mi3 (Addgene ID: 113043) plasmids were a kind gift from Professor Mark Howarth’s group at the University of Oxford, UK. Plasmid pTJDNA4, which expresses HIV-1 subtype C mosaic Gag [26], was described previously. Goat anti-HIV-1 gp120 (Bio-Rad, Hercules, CA, USA 5000-0557), goat anti-HIV-1 p24 (Gag) (Bio-Rad, 4999-9007), mouse monoclonal anti-goat/sheep IgG-alkaline phosphatase (AP) GT-34 (Sigma-Aldrich, St. Louis, MI, USA, A8062), mouse anti-histidine tag (Bio-Rad, Hercules, CA, USA formerly Serotech, MCA1396) and goat anti-mouse IgG-alkaline phosphatase (Sigma-Aldrich, St. Louis, MI, USA, A3562) were used for Western blotting. Anti-HIV-1 Env human monoclonal antibodies (MAbs) PG16, PGT128, PGT145, CAP256-VRC26.08, VRC01, 10E8 and 447-52D were used. The MAbs were expressed in FreeStyle^TM^ 293-F cells (ThermoFisher Scientific, Waltham, MA, USA) using the PEI MAX^®^ transfection reagent (Polysciences, Warrington, PA, USA) and purified from cell-free supernatants after 6 days using protein A affinity chromatography. HEK293 (ATCC^®^, CRL-1573^TM^), HEK293T (ATCC^®^, CRL-3216^TM^) and HeLa (ATCC^®^, CCL-2^TM^) cells were cultured in Dulbecco’s modified Eagle medium (DMEM) with high glucose supplemented with L-glutamine, 10% foetal calf serum (both Gibco, ThermoFisher Scientific, Waltham, MA, USA) and 1× penicillin-streptomycin (Pen-Strep) (Lonza, Basel, Switzerland). Escherichia coli BL21-CodonPlus (DE3)-RIL cells (Agilent Technologies, Santa Clara, CA, USA) were used to express SpyCatcher mi3 and MBP SpyTag.

### 2.2. CAP255 gp140 SpyTag & gp150 Design

**Figure 1:**
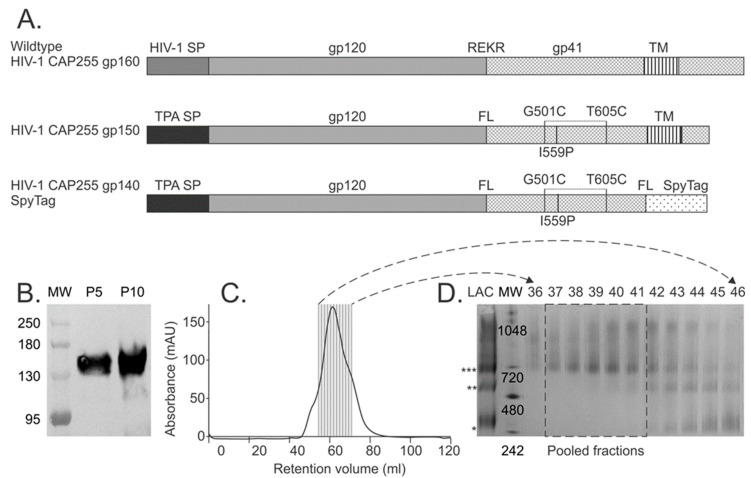
Design and characterisation of HIV-1 CAP255 Env. (**A**) Schematic diagram of the original wildtype HIV-1 CAP255 gp160, the truncated CAP255 gp150 and the soluble CAP255 gp140 SpyTag. HIV-1 SP—signal peptide; REKR—furin cleavage site; TM—transmembrane region; TPA SP-tissue plasminogen activator leader sequence; FL-flexible linker; G501C—glycine-to-cysteine mutation at amino acid 501; T605C—threonine-to-cysteine mutation at amino acid 605; I559P—isoleucine-to-proline mutation at amino acid 559. (**B**) Western blot confirming expression of CAP255 gp140 SpyTag in the media of stably transfected HEK293 cells at passage 5 (P5) and 10 (P10). MW—molecular weight marker. (**C**) Graph showing the SEC profile of CAP255 gp140 SpyTag and the fractions analysed in (**D**) are shown. (**D**) Coomassie-stained Blue Native PAGE of CAP255 gp140 SpyTag purified by lectin affinity chromatography (LAC) and subsequent SEC fractions 36 to 46. CAP255 gp140 SpyTag trimers (***), dimers (**), monomers (*) and molecular weight in kDa (MW) are indicated.

The HIV-1 Env sequence used in this study was derived from patient CAP255, a participant from the CAPRISA 002 Acute Infection 2004 study, a cohort of HIV-1-subtype-C-infected women who developed broadly cross-neutralizing antibodies against HIV-1 [27,28]. The wildtype envelope (Env) gp160 sequence of a virus isolated eight weeks post-infection was modified as follows (Figure 1A): the native signal peptide sequence (HIV-1 SP) was replaced with that of the human tissue plasminogen activator (TPA), the furin cleavage site (KEKR) was replaced with a flexible linker (FL) (GGGGSGGGGS) [29,30], G501C and T605C mutations were introduced and an I559P mutation was introduced in gp41 [31]. The Env was truncated to gp150 for the DNA vaccine and gp140 for the soluble protein. In addition, the SpyTag sequence (AHIVMVDAYKPTK) was fused to the C-terminus of the gp140 using a short FL sequence [32]. The modified Env sequences were human codon optimised and synthesised by GenScript, Piscataway, NJ, USA. The gene inserts were cloned into our pMExT (pTHpCapR) mammalian expression vector [33] to generate pMExT-CAP255 gp150 and pMExT-CAP255 gp140 SpyTag. Plasmid pMExT-CAP255 gp140 SpyTag IRES Neo was constructed by insertion of the internal ribosome entry site (IRES)-neomycin directly downstream of the SpyTag in plasmid pMExT-CAP255 gp140 SpyTag.

### 2.3. CAP255 gp150 Expression and Characterisation

HEK293T cells were transfected with pMExT CAP255 gp150 and pJTDNA4. The cell lysate and media were isolated at 72 h post-transfection and used for Western blotting as described in van Diepen et al. [34]. Immunostaining of HeLa cells transfected with pMExT-CAP255 gp150 or pMExT-CAP256 gp150 (control) with MAbs was carried out to assess the tertiary structure of the CAP255 gp150 [34].

### 2.4. CAP255 gp140 SpyTag Expression, Isolation and Characterisation

A stable cell line expressing CAP255 gp140 SpyTag was generated following transfection of the HEK293 cells with the pMExT-CAP255 gp140 SpyTag IRES Neo plasmid. The cells were passaged at least 10 times in a selection medium containing 250 μg/mL geneticin (Gibco). Soluble CAP255 gp140 SpyTag was isolated from the serum-free media using a two-step purification method: agarose lectin–mannose affinity column from Galanthus nivalis snowdrop plants (Sigma-Aldrich, St. Louis, MO, USA) and size exclusion chromatography (SEC) HiLoad™ 16/600 Superdex 200 pg gel filtration column (GE Healthcare, Chicago, Illinois, USA) to fractionate by size as described previously [34]. The fractions containing CAP255 gp140 SpyTag trimers were pooled together, concentrated, then aliquoted for storage at −80 °C. The protein concentration was measured using a DC protein assay (Bio-Rad, Hercules, CA, USA) as per the manufacturer’s protocol.

The expression of CAP255 gp140 SpyTag in the HEK293/HEK293T cells was characterised by denaturing SDS polyacrylamide gel electrophoresis (SDS-PAGE) and Western blotting using 1:1000 goat anti-gp120, followed by 1:10,000 mouse monoclonal anti-goat/sheep IgG-AP. The SEC-purified CAP255 gp140 SpyTag fractionates were characterised by resolving on precast Blue NativePAGE Novex 3–12% Bis-Tris Protein Gels (Life Technologies, Carlsbad, CA, USA), which were stained with Bio-Safe Coomassie (Bio-Rad, Hercules, CA, USA).

### 2.5. Expression of SpyCatcher mi3 and MBP SpyTag Proteins

pET28a-SpyCatcher mi3 and pET28a-MBP SpyTag plasmids were transformed into competent *E. coli* BL21-CodonPlus (DE3)-RIL cells (Agilent Technologies, Santa Clara, CA, USA) and expressed as detailed in [13,35]. In short, the *E. coli* BL21 clones were grown on Luria Bertani (LB) agar plates supplemented with 50 µg/mL kanamycin and incubated at 37 °C overnight. A single colony of each recombinant clone was picked to inoculate 10 mL starter cultures of LB broth supplemented with 50 µg/mL kanamycin and incubated at 37 °C on a shaker platform (200 rpm) overnight. The starter culture was then diluted into 1000 mL LB broth supplemented with 0.8% glucose and 50 µg/mL kanamycin; the culture was incubated on an orbital shaker until the optical density at 600 nm (OD_600_) reached 0.5–0.6. Protein expression was induced with 0.42 mM free isopropyl ß-D-1-thiogalactopyranoside (IPTG) (Sigma-Aldrich, St. Louis, MO, USA). The SpyCatcher mi3 culture was incubated further at room temperature for 16 h, whilst the MBP SpyTag was incubated at 30 °C for 4 h only. The cultures were harvested by centrifugation at 4000× *g* (Sorvall, Waltham, MA, USA, RC5B Plus) at 4 °C for 10 min.

### 2.6. Purification of SpyCatcher mi3 NPs

The pelleted cells were frozen for 2 h at −20 °C and resuspended in 5 mL/g of binding buffer [20 mM NaH_2_PO_4_, 0.5 mM NaCl, 40 mM Imidazole pH 7.4] supplemented with 1× cOmplete mini EDTA-free protease inhibitor (Roche, Basel, Switzerland). The cell suspension was sonicated on ice for 4 min, 15 s on and 15 s off pulsations at 85% power using the liquid processor probe (Misonix^®^ Sonicator 3000 Ultrasonic). The lysed sample was clarified at 16,000× *g* at 4 °C for 30 min (Sorvall, Waltham, MA, USA RC5B Plus) and filtered through a 0.45 µm syringe filter. The SpyCatcher mi3 nanoparticles were purified using a prepacked HisTrap™ HP 5 mL column (Cytiva, Marlborough, MA, USA) on the AKTAExplorer fast protein liquid chromatography (FPLC) system at UV280 nm. The clarified lysate was loaded into the sample super loop at a 5 mL/minute rate; afterwards, the column was washed with 8 column volumes of the binding buffer until the UV light absorbance reading stabilised. The bound protein was eluted with 5 column volumes of 100% elution buffer [20 mM NaH_2_PO_4_, 0.5 mM NaCl, 500 mM Imidazole pH 7.4]. To remove aggregates, 5 mL of the concentrated eluate was loaded onto a High Prep™ 16/60 Sephacryl^®^ S-500 HR column (Sigma-Aldrich, St. Louis, MO, USA) to fractionate at the applied flow rate of 0.5 mL/minute using 1× phosphate buffer saline (PBS) pH 7.4. The SpyCatcher mi3 NPs were aliquoted and stored at −80 °C.

### 2.7. Purification of MBP SpyTag

The pelleted cells were resuspended in 20 mL of binding buffer [50 mM Tris-HCl, 5 mM Imidazole, 100 mM NaCl, 0.1 mM EDTA, pH 8] supplemented with 0.1 mg/mL lysozyme, 1 mM phenylmethanesulfonyl (PMSF) (both from Sigma-Aldrich, St. Louis, MO, USA) and 1 mg/mL cOmplete mini EDTA-free protease inhibitor (Merck, Rahway, NJ, USA). The cell suspension was sonicated on ice 5× times in 60 s cycles, with 20 s breaks between 50% duty-cycle (Virtis, Los Angeles, CA, USA, VirSonic Cell Disrupter model 16-1850). The sample was clarified at 17,000× *g* at 4 °C for 15 min (Sorvall, Waltham, MA, USA, RC5B Plus), and the supernatant was chilled on ice for ~30 min before further purification steps. Ten millilitres of the HisPur™ Cobalt resin (ThermoFisher Scientific, Waltham, MA, USA) were washed with the binding buffer as per manufacturer protocol, mixed with the lysate and incubated on a rotary shaker for one hour. The prepared sample was loaded onto a 10 mL cottonwool-plugged gravity flow column. The collected flowthrough was reapplied twice, and the resin was washed with five column volumes of the wash buffer [50 mM Tris-HCl, 10 mM Imidazole, 300 mM NaCl, 0.1 mM EDTA, pH 8]. The bound proteins were eluted with 20 mL of the elution buffer [50 mM Tris-HCl, 300 mM Imidazole, 50 mM NaCl, 0.1 mM EDTA, pH 8], collected in ~800 μL fractions and quantified by OD_280_ readings. The fractions were pooled and loaded onto a Slide-A-Lyzer^®^ 10 kD MW cut-off membrane (ThermoFisher Scientific, Waltham, MA, USA formerly Pierce) and dialysed overnight in 500-fold excess volume of 1× PBS at 4 °C to remove excess imidazole.

### 2.8. SpyCatcher mi3: CAP255 gp140 SpyTag/MBP SpyTag Conjugation Reactions

The CAP255 gp140 SpyTag trimers or MBP SpyTag were conjugated to the SpyCatcher mi3 NPs at different molar ratios in vitro to optimise coupling. The coupled reactions were incubated at 4 °C overnight in TBS reaction buffer [25 mM Tris-HCl, 300 mM NaCl, pH 8.5] [16]. The MBP SpyTag coupling reactions were resolved by SDS-PAGE/Coomassie staining, whereas the CAP255 gp140 SpyTag reactions were first deglycosylated using peptide N-glycosidase (PNGase F) (New England Biolabs, Ipswich, MA, USA). Deglycosylation was performed to remove the N-linked glycans on the glycoproteins to improve the band resolution on SDS-PAGE/Western blotting. Following protein detection, the band intensities on the blots were quantified using Imager^®^ Gel Doc™ XR software (6.1.0 build 7, Bio-Rad, Hercules, CA, USA) to estimate the coupling efficiency.

### 2.9. Negative Stain Electron Microscopy (NS-EM) Data Collection

The SpyCatcher mi3 NPs, MBP SpyTag–SpyCatcher mi3 NPs, CAP255 gp140 SpyTag-and SpyCatcher mi3 NPs (without PNGase F) were pipetted onto freshly glow-discharged carbon-coated grids at 25 mA (EMS100 Glow Discharge Unit) (Electron Microscope Sciences, Hatfield, PA, USA), washed three times with distilled water and stained with two changes of 2% uranyl acetate (SPI Supplies, West Chester, PA, USA). Excess liquid was blotted off with filter paper between each rinsing and staining step, with the final blot leaving a thin layer of stain, which was allowed to dry at room temperature before imaging [36]. The data (3.12 Å/pixel) were collected using a FEI Tecnai 20 transmission electron microscope (ThermoFisher Scientific, Waltham, MA, USA formerly FEI) operating at 200 kV (Lab6 emitter) and a CCD camera (Gatan, Pleasanton, CA, USA, US1000).

### 2.10. NS-EM Image Processing

For each dataset, ~100 particles were manually picked, extracted into 256 × 256 pixel boxes and used to generate 2D class averages. A single class was used for auto-picking, yielding an initial dataset. Poorly aligned particles were eliminated via 2D classification. De novo initial 3D models were created via stochastic gradient descent while imposing icosahedral symmetry [37]. The models were masked, refined and postprocessed to yield ~20 Å resolution maps. All the image processing steps were performed using RELION V3.1 [38].

### 2.11. Cryogenic Electron Microscopy (Cryo-EM) Data Collection

CAP255 gp140 SpyTag–SpyCatcher mi3 NPs (2.5 μL diluted 1:10) were applied to glow-discharged 2/2 Quantifoil holey carbon grids (Quantifoil Micro Tools GmbH, Jena, Germany) overlaid with a layer (2–3 nm thick) of continuous amorphous carbon. After application, the grid was immediately blotted (4 °C, 100% humidity, t = 3.5 s force = 0) and vitrified by plunging into slushy ethane [39] using a Vitribot IV (ThermoFisher Scientific, Waltham, MA, USA formerly FEI). The grids were screened using a Tecnai F20 microscope (ThermoFisher Scientific, Waltham, MA, USA formerly FEI) with a DE16 direct detector (Direct Electron, San Diego, CA, USA) at 200 kV, and the data were collected using a Titan Krios microscope (ThermoFisher Scientific, Waltham, MA, USA) at the electron Bio-Imaging Centre (eBIC) at Diamond Light Source using a nominal magnification of 105 k× and 300 kV with a K3 detector (Gatan, Pleasanton, CA, USA, US1000) in super-resolution mode at a 2× downsampled pixel size of 0.831 Å.

### 2.12. Cryo-EM Image Processing

All the image processing steps were performed with Relion 4.0.1 [38]. A total of 27,642 fifty-frame movies were collected and aligned with MotionCor2 [40], and defocus was estimated with CTFFIND 4.1 [41]. Two-dimensional class averages were generated with ~1000 manually selected particles and used as auto-picking templates to yield ~500,000 particles. These were refined via several rounds of 2D and 3D classification to a final set of 1439 particles, which were used to produce an initial icosahedral map via Stochastic Gradient Descent [37] and refined to a resolution of 5.4 Å after β-factor correction and masking of the CAP255 gp140 SpyTag trimer densities (SpyCatcher mi3 NP map). The particles were then re-extracted into a larger box size to accommodate the CAP255 gp140 SpyTag trimer densities, and the entire icosahedral NP was refined to a resolution of 9.4 Å without masking (CAP255 gp140 SpyTag–SpyCatcher mi3 NP map). A mask covering a single CAP255 gp140 SpyTag trimer density was made from this map using UCSF Chimera 1.16 [42] and refined through cycles of 3D classification without symmetry imposition and increasing the value of “T” up to T = 40, yielding a reconstruction at a resolution of 17.7 Å (CAP255 gp140 SpyTag–SpyCatcher map).

### 2.13. Model Building, Interpretation and Visualisation

The structures of the SpyCatcher003 mi3 (PDB ID: 7B3Y) [21], SpyCatcher/SpyTag (PDB ID: 4MLS) [43] and BG505 SOSIP.664 gp140 trimer (PDB ID: 5JSA) [44] were downloaded from the Electron Microscopy Data Bank (https://www.ebi.ac.uk/emdb/) or Protein Data Bank (https://www.rcsb.org/) (accessed on 25 January 2021) and docked as rigid bodies into the EM maps. SpyCatcher mi3 was modelled using the AlphaFold 3 prediction server [45], and the resulting structure was docked using Isolde 1.6 [46] in ChimeraX 1.7.1 [47]. Visualisation of the 3D reconstructions and atomic resolution coordinates, as well as rendering of the images, was conducted using UCSF Chimera 1.16 [42].

### 2.14. Rabbit Immunisations

Female New Zealand White rabbits (age ±10 weeks, weight ≥ 2.2 kg) were housed in the Faculty of Health Sciences animal facility at the University of Stellenbosch. All the animal procedures were approved by the UCT Animal Research Ethics Committee (reference UCT AEC 019-015) and performed by trained animal technologists. DNA vaccines were administered intramuscularly into the hind leg at 100 µg (100 µL of each). The DNA vaccines consisted of two plasmids, formulated together in equal quantities with CpG adjuvant ODN 1826 (27.5 μg/rabbit, Miltenyi Biotec, Bergisch Gladbach, Germany); the first expressed the HIV-1 CAP255 gp150 envelope protein, and the second, the subtype C mosaic Gag (100 µg of each). The DNA vaccines were administered with the PharmaJet Stratis (PharmaJet, Golden, CO, USA) device as indicated. The Env SpyTag trimers were conjugated to SpyCatcher mi3 NPs at a 1:1 molar ratio at 4 °C overnight. The following day, the conjugated NPs were mixed with an equal volume of Alhydrogel adjuvant, and the rabbits were immunised intramuscularly with the equivalent of 50 µg of Env (500 µL). The animals were bled at weeks 0, 6, 14, 22 and 30. The week 0 bleeds were used as prebleeds.

### 2.15. Anti-Env Binding Titres

Env-binding antibody titres in the rabbit sera were determined as follows. Briefly, Nunc MaxiSorp^®^ flat-bottom 96-well plates (Sigma-Aldrich, St. Louis, MO, USA) were coated with 10 ng/well soluble, trimeric CAP255 Env overnight. Rabbit sera were used in the primary incubation in a serial dilution range starting at 1:10. Anti-rabbit IgG HRP (1:5000, Roche, Basel, Switzerland) was used for detection with TMB ELISA Substrate (Abcam, Cambridge, UK). The reaction was stopped after 10 min with 1N H_2_SO_4_. The ELISA signal was analysed using a VersaMax ELISA Microplate Reader (Molecular Devices, San Jose, CA, USA), which subtracted absorbance values at 540 nm from values at 450 nm. ELISAs for the whole-time course were performed at the same time on duplicate plates. The duplicate data points were averaged and fitted to a four-parameter logistic regression curve (4PL curve) in GraphPad Prism 8.0. Antibody end-point titres were calculated from the 4PL curves, with the threshold set as 4PL curve minimum + standard error of minimum for each time point. The data were plotted as the mean +/− SEM for the whole group.

### 2.16. Pseudovirus-Based Neutralisation Assays

The standardised TZM-bl pseudovirus neutralisation assay [48] was used to determine the neutralising antibody titres. Briefly, neutralisation was measured as a reduction in luciferase gene expression after a single round of infection of TZM-bl cells (NIH AIDS Research and Reference Reagent Program) with Env-pseudotyped viruses. The titres were calculated as the reciprocal serum dilution, causing a 50% reduction in the relative light units (ID50). The dilutions were started at 1:40. MuLV was used as a negative control.

## 3. Results

### 3.1. Design, Expression and Characterisation of CAP255 gp140 SpyTag and gp150 Proteins

The envelope sequence used in this study was derived from a virus isolated eight weeks post-infection from a patient in the South African CAPRISA 002 acute infection cohort, patient CAP255, who developed broadly neutralising antibodies targeting the N332 epitope [27,28]. The sequence was modified to improve expression and stability as follows: the native leader sequence was replaced with the tissue plasminogen leader sequence (TPA) to enhance secretion, the furin cleavage site was replaced with a flexible linker sequence (GGGGSGGGGS) [29,30], G501C and T605C mutations were introduced to stabilise the linkage between gp120 and gp41 with a disulphide bond and an I559P mutation was introduced to improve trimerisation of gp41 (Figure 1A) [31]. The envelope sequence was truncated to amino acid 652 to generate soluble gp140 protein, and the SpyTag sequence was fused to the C-terminus with a short linker sequence. For the DNA vaccine, the gp160 sequence was truncated to gp150 (amino acid 730) to retain the transmembrane domain for presentation on the surface of Gag virus-like particles (Figure 1A).

A stably transfected HEK293 cell line expressing CAP255 gp140 SpyTag was generated, and Western blotting (~145 kDa) confirmed protein expression (Figure 1B). The trimeric fraction was purified using a two-step method consisting of lectin-affinity chromatography followed by SEC (Figure 1C,D). Fractions 37–41 (55.5 mL–63 mL) (Figure 1C), which consisted mainly of trimers (~720 kDa) with some aggregates (>720 kDa), were pooled and used in coupling reactions with SpyCatcher mi3 NPs.

**Figure 2:**
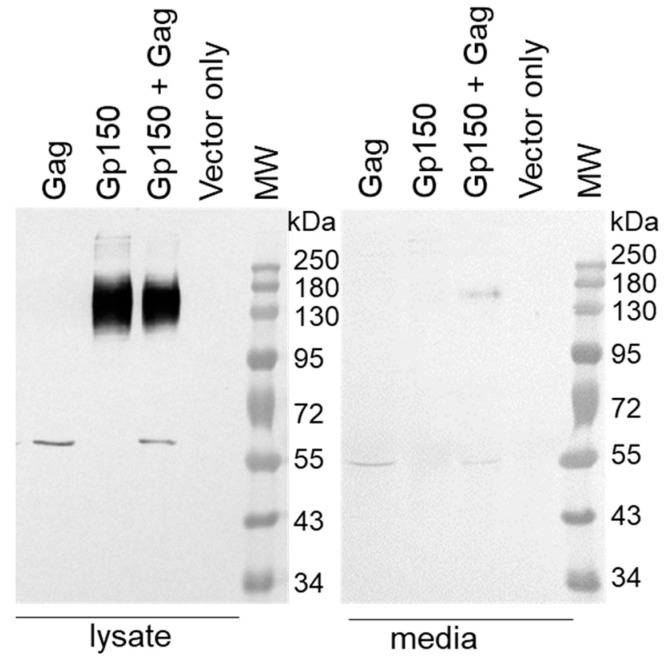
Western blotting confirming the expression of CAP255 gp150 Env and mosaic Gag.

Western blotting confirmed the expression of Env (gp150) and Gag by HEK293T cells co-transfected with pMExT-CAP255 gp150 and pTJDNA4 (Figure 2). Env and Gag were detected in the cell media, indicating that both proteins are secreted. Previous work by our group has shown that cells co-transfected with pTJDNA4 and a DNA vaccine expressing CAP256SU gp150 formed Gag VLPs containing Env [32], so it is likely that cells transfected with DNA vaccines expressing Gag and CAP255 gp150 also form VLPs, although this was not confirmed in this study.

Immunostaining with human monoclonal binding antibodies (MAbs) to HIV-1 Env, followed by fluorescent microscopy (Table 1, Figure S2), was carried out to assess the structure of gp150 expressed on the cell surface. CAP256SU gp150 was included as a control [34]. Both the CAP255 and CAP256 gp150 envelope proteins were bound by neutralising MAbs PGT128 (V3 glycan), VRC01 (CD4 binding site), F105 (CD4 binding site), 10E8 (MPER) and 447-52D (V3-specific, non-neutralising) at similar levels. The CAP256 gp150 bound MAbs PG9, PGT145 and CAP256-VRC26.08, which bind to the V1/V2 epitope at the tip of the Env trimer. The CAP255 gp150 showed no or little binding of MAbs PG9, PGT145 and CAP256-VRC26.08.

### 3.2. Preparation and Coupling of Env-Nanoparticles

**Figure 3:**
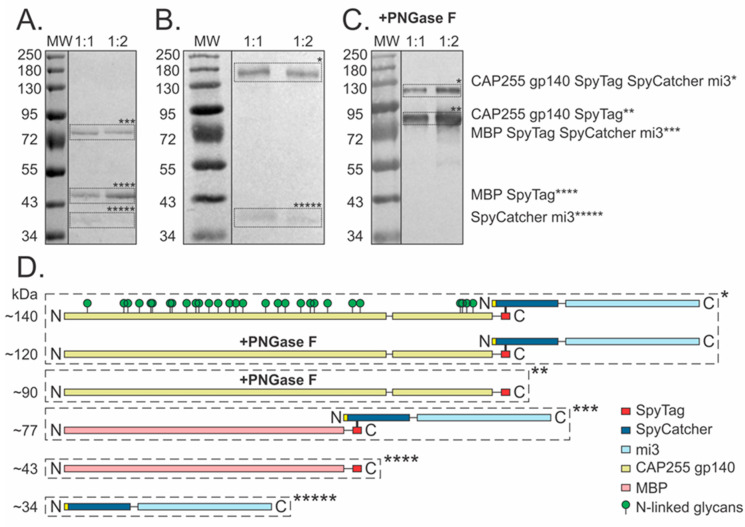
SpyTag–SpyCatcher coupling efficiency. (**A**) Coomassie-stained SDS-PAGE analysis of coupling reaction with MBP SpyTag**** and SpyCatcher mi3***** NPs at 1:1 and 1:2 molar ratios. Doubling the concentration of MBP SpyTag increased the amount of unbound MBP SpyTag****, and only a negligible amount of unbound SpyCatcher mi3***** remained. (**B**) Western blot (anti-His tag) analysis of CAP255 gp140 SpyTag:SpyCatcher mi3* coupling reaction. Doubling the concentration of CAP255 gp140 SpyTag had no noticeable effect on the amount of CAP255 gp140 SpyTag SpyCatcher mi3*, as unbound SpyCatcher mi3***** was still clearly visible. (**C**) Western blot (anti-Env) showing a large excess of CAP255 gp140 SpyTag** and an increase in CAP255 gp140 SpyTag SpyCatcher mi3* after doubling the concentration of CAP255 gp140 SpyTag** in the coupling reaction. (**D**) Diagrammatic representation of the proteins detected.

The self-assembling 60-subunit SpyCatcher mi3 nanoparticles were expressed in *E. coli* BL21 and purified using a prepacked nickel affinity (Ni-NTA) HisTrap™ column. To confirm that the SpyTag–SpyCatcher system was functional, MBP SpyTag was coupled to SpyCatcher mi3, and approximately 100% coupling efficiency was achieved (~60 MBP monomers could be displayed on the surface of the mi3 NP) (Figure 3A). Unlike MBP, HIV Env proteins resolve very poorly due to the high and variable glycosylation, resulting in hazy and diffuse bands [49]. To avoid this, the samples of coupled CAP255 gp140 SpyTag–SpyCatcher mi3 were first treated with PNGase F, a deglycosylation reagent, to remove the exposed N-linked glycans prior to blotting (anti-Env). Although SDS-PAGE/Coomassie is a preferred method to analyse coupling efficiency, due to the similar size between PNGase F (~36 kDa) and the SpyCatcher mi3 NPs (~34 kDa), Western blotting (anti-His tag) was used to assess the coupling of CAP255 gp140 SpyTag to the SpyCatcher mi3 NPs.

**Figure 4:**
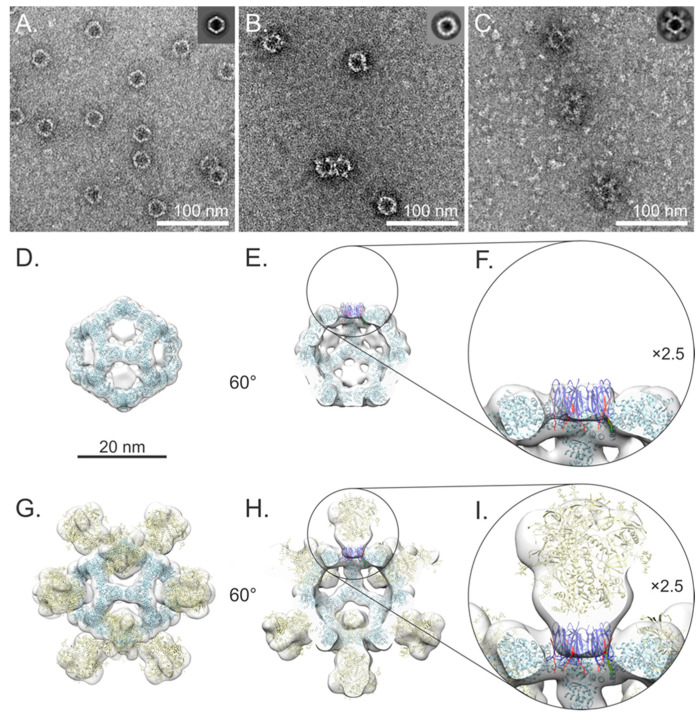
NS-EM of the SpyCatcher mi3 NPs coupling. (**A**) SpyCatcher mi3 NPs and 2D class average (inset) showing successful assembly. (**B**) MBP SpyTag–SpyCatcher mi3 NPs; MBP densities make the SpyCatcher mi3 wall appear slightly thicker. (**C**) CAP255-gp 140 SpyTag–SpyCatcher mi3 NPs; excess Env particles can be seen in the background. Clear additional density corresponding to externally protruding Env trimers can be seen on the surface of the NP in the raw images and 2D class average (inset). The slight blurring of Env trimers, visible in the 2D class average, is likely due to conformational flexibility. (**D**) Three-dimensional map of SpyCatcher mi3 NP docked with the atomic coordinates of mi3 (PDB ID: 7B3Y in light blue), demonstrating good particle assembly. Additional density was observed at the pentagonal NP faces. (**E**,**F**) In negative stain, the details of this additional density could not be resolved, but its dimensions and shape correspond closely to five copies of SpyCatcher protein (PDB ID: 4MLS in dark blue). (**G**) CAP255 gp140 SpyTag–SpyCatcher mi3 NP reconstruction; the mi3 NP (PDB ID: 7B3Y in light blue) maintains rigidity and is surrounded by twelve regularly spaced Env trimers with high occupancy (36 copies of CAP255 gp140 SpyTag). The atomic structures of the Env trimer (PDB ID: 5JSA in yellow) correspond closely to the contours of the 3D map and are aligned along the five-fold axis. The clear 5-fold symmetry of the trimers is an artefact of imposing icosahedral symmetry on the trimer at the icosahedral 5-fold axes. (**H**,**I**) An additional disk of density corresponding to (**D**) was observed at the base of each Env trimer.

The coupling efficiency was assessed by altering the ratio of CAP255 gp140 SpyTag to SpyCatcher mi3 (Figure 4B,C). Increasing the proportion of CAP255 gp140 SpyTag beyond a 1:1 molar ratio did not appear to increase the amount of coupled product or decrease unbound SpyCatcher mi3 (Figure 3B). As expected, treating CAP255 gp140 SpyTag reactions with PNGase F decreased the molecular weight from ~140 kDa to ~90 kDa (Figure 3C). Increasing the amount of CAP255 gp140 SpyTag in the coupling reaction did not lead to a concomitant increase in the mi3-Env NPs. However, a significant increase in the amount of uncoupled Env-SpyTag was seen (Figure 3C). A molar ratio of 1:1 mi3: Env was selected for further experiments, as it generated a reasonable amount of CAP255 gp140 SpyTag SpyCatcher mi3 NPs while minimising uncoupled Env and mi3.

### 3.3. NS-EM Structural Analysis

Negative staining TEM analysis confirmed the presence of homogenous and stable mi3 particles. These were imaged again after coupling to confirm the SpyTag-mediated modular display of MBP and CAP255 gp140 on their surfaces. The unconjugated SpyCatcher mi3 NPs assembled into ~25 nm hollow spherical particles with distinct icosahedral symmetry after 2D classification (Figure 4A). Following coupling with MBP SpyTag (~34 kDa), the SpyCatcher mi3 NP diameter increased slightly (Figure 4B). After coupling with trimeric, ~720 kDa, CAP255 gp140 SpyTag, the SpyCatcher mi3 NPs were surrounded by regularly spaced outwardly projecting densities, which were revealed to be symmetrically arranged after 2D classification. Excess uncoupled trimeric Env is visible in the background as globular trimeric structures (Figure 4C).

A NS-EM 3D reconstruction of the SpyCatcher mi3 NPs in Relion [38] confirmed that the NPs assembled into stable dodecahedral “cages” with icosahedral symmetry (Figure 4D). The data collection, refinement and validation statistics are shown in (Table S1). Disks of additional density were observed at the pentagonal faces of the mi3 NP (Figure 4C,D). To investigate if this could be attributed to SpyCatcher, we docked five copies of the X-ray crystallographic structure of SpyCatcher (PDB ID: 4MLS) [43] into one of the disks and found that both the overall size and shape agreed (Figure 4 E,F). The NS-EM 3D map of the CAP255-gp140 SpyTag–SpyCatcher mi3 NPs shows a distinct undistorted dodecahedral cage with the same size and shape as the SpyCatcher mi3 NP but surrounded by additional protein densities projecting outwards from each of the twelve pentagonal faces of the dodecahedral mi3 NP (Figure 4G). These were fitted with the X-ray crystallographic structure of the gp140 trimer (PDB ID: 5JSA) [44], which showed the same overall shape and size (Figure 4G–I). In the cross-section, additional density is visible between the CAP255 gp140 SpyTag density and SpyCatcher mi3 NP; this superimposes precisely on the density visible at the pentagonal face of the SpyCatcher mi3 dodecamer (Figure 4F,I) and which we propose corresponds to five SpyCatcher subunits.

### 3.4. Cryo-EM Single Particle Analysis

Cryo-EM shows the native conformation of proteins without drying- and staining artefacts and has the potential to achieve better resolution than NS-EM. To confirm our preliminary structural results and further interrogate the structure of CAP255 gp140 SpyTag–SpyCatcher mi3, we attempted a cryo-EM experiment on the assembled NPs (Table S1). A preliminary screening showed that the particles aggregated along the periphery of the grid-holes, an effect that was improved by overlaying the grids with a ~2–3 nm layer of amorphous carbon and lowering the protein concentration. In total, ~28,000 micrographs were collected over a 48 h period. Image processing was performed in Relion 4.01 (Scheres, 2016) to maximise both the number and quality of particles. A low-threshold was used during automatic particle picking, and poor-quality images were eliminated by multiple rounds of 2D- and 3D classification. This process yielded 1439 particles, which were used for subsequent analysis.

**Figure 5:**
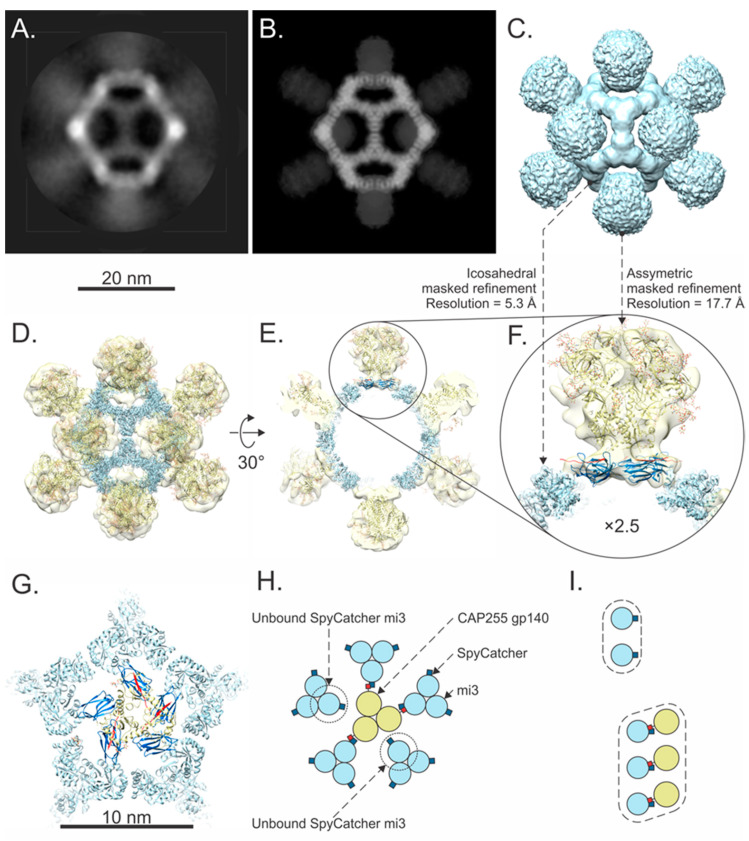
Cryo-EM analysis of the assembled nanoparticles. (**A**) Two-dimensional class average of the CAP255 gp140 SpyTag SpyCatcher mi3 NP visualised along the icosahedral 2-fold symmetry axis. The stable mi3 core is surrounded by flexible CAP255 gp140 densities. (**B**) Density view of the icosahedral 3D reconstruction, rotated to the same orientation as (**A**). (**C**) Surface representation of the reconstruction; Env densities are centred on the 5-fold icosahedral symmetry axes. (**D**) Masked and symmetry-imposed mi3 NP density (blue) at 5.3 Å resolution, and CAP255 gp140 SpyTag–SpyCatcher density (yellow) at 17.7 Å resolution after focused classification without imposing symmetry. The atomic coordinates of mi3 (PDB ID: 7B3Y in light blue), SpyCatcher/SpyTag (PDB ID: 4MLS in dark blue and SpyTag in red) and the gp140 trimer (PDB ID: 5JSA yellow) are shown docked into the 3D reconstructions. (**E**,**F**) Five SpyCatcher monomers associate symmetrically about the five-fold axis to form a disk. The points of attachment to mi3 can be seen. Three-fold symmetric CAP255 gp140 SpyTag binds to the SpyCatcher disk. (**G**–**I**) The association between CAP255 gp140 SpyTag and SpyCatcher mi3 visualised along the icosahedral 5-fold symmetry axis shows the symmetry mismatch between the CAP255 gp140 SpyTag trimer and SpyCatcher pentamer. This results in a ratio of 3:2 (covalently linked SpyCatcher mi3: unlinked SpyCatcher mi3).

The 2D-classified averages showed a stable mi3 NP core (Figure 5A) surrounded by more flexibly arranged CAP255 gp140 Env trimers (Figure 5A). Reconstructing the particle in 3D using a large mask diameter (equal to the box size) and imposing icosahedral symmetry yielded a map with an average resolution of 9.4 Å, composed of an NP surrounded by twelve weaker CAP255 gp140 Env trimer densities (Figure 5B,C). To improve the overall resolution, this 3D reconstruction was manually edited in UCSF Chimera [42] to exclude Env and used to create a mask in Relion 4.01 [38]. Refinement using this mask yielded an mi3-NP-only map at a resolution of 5.4 Å after β-factor correction (Figure 5D–F), allowing good visualization of the secondary structure and precise location of mi3 atomic coordinates (PDB ID: 7B3Y). This demonstrated unequivocally that the mi3 NP protein subunits were not distorted relative to the atomic coordinates. A second map was reconstructed by masking out all but a single CAP255 gp140 Env trimer during 3D classification without imposing symmetry. A regularisation parameter (T) = 20 led to a 17.7 Å resolution reconstruction (Figure 5D–F).

Pentameric SpyCatcher (Figure 5F) and its connections to mi3 are visible in this reconstruction (Figure 5F,G). The resulting symmetry mismatch means that for every five mi3 SpyCatcher subunits, up to three are available to bind CAP255 gp140 SpyTag, while the remaining two remain unbound (Figure 5G–I).

### 3.5. Rabbit Immunogenicity

**Figure 6:**
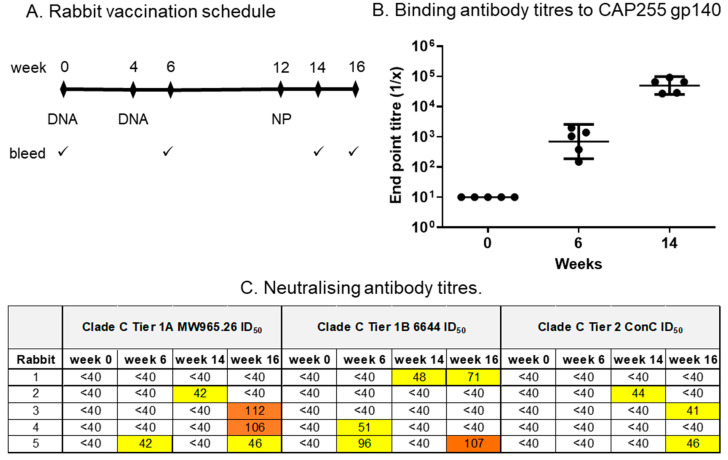
Binding and neutralising antibody responses in rabbits. (**A**) Vaccination schedule and bleeds. (**B**) Binding antibody titres to CAP255 gp140 in sera of vaccinated rabbits. Where no binding was observed, the endpoint titre was plotted as 10. (**C**) Neutralising antibody titres in rabbit sera were measured using the pseudovirus-based TZM-bl neutralisation assay. The serum was taken 2 weeks after the second DNA prime (week 6), 2 weeks after the nanoparticle inoculation (week 14) and 4 weeks after the nanoparticle inoculation (week 16). Neutralisation titres were negative for all time points in the MuLV negative-control neutralisation assay. The 50% neutralisation titres (ID50) are colour-coded to reflect their potency range. Titres below 40 were considered non-neutralising and were not colour-coded.

**Figure 7:**
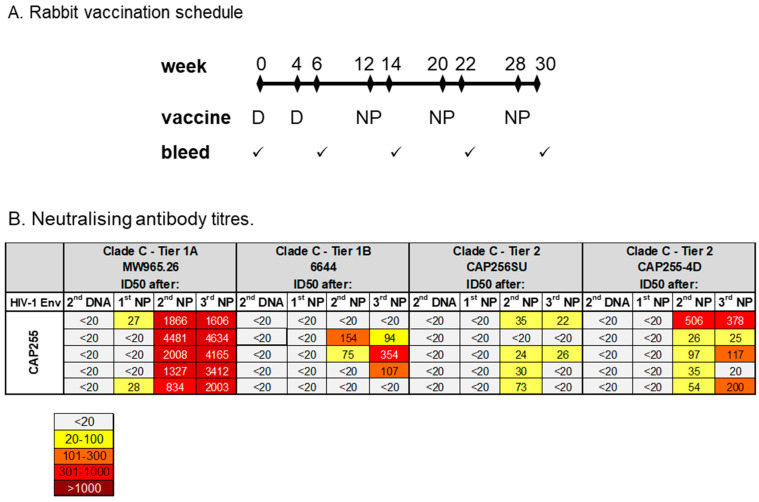
Neutralising antibody responses in rabbits. (**A**) Vaccination schedule and bleeds. (**B**) Neutralising antibody titres in rabbit sera were measured using the TZM-bl assay. The serum was taken 2 weeks after the second DNA prime (2nd DNA) and 2 weeks after each nanoparticle inoculation (1st NP, 2nd NP and 3rd NP). Neutralisation titres were negative for all time points in the MuLV negative-control neutralisation assay. The 50% neutralisation titres are colour-coded to reflect their potency range. Titres below 20 were considered non-neutralising and were not colour-coded.

To determine whether two DNA vaccine immunisations followed by a single dose of nanoparticles elicited a good neutralising antibody response, the rabbits were inoculated with two doses of the DNA vaccines mixed with CpG adjuvant (Env+Gag, 100 µg of each) at weeks 0 and 4, followed by a single dose of nanoparticles at week 12 (Figure 6A). A DNA prime/nanoparticle boost protocol was used, as previous reports have shown that DNA priming strongly favours the development of both humoral and cellular responses following a heterologous boost [26,34,50,51]. The rabbits were bled at the time points shown in Figure 6A, and the immunogenicity was assessed using an ELISA of the CAP255 Env trimer and neutralisation assays. All five rabbits developed binding antibodies after the second DNA inoculation, which were strongly boosted following vaccination with the nanoparticles (Figure 6B). Three of the five rabbits developed neutralising antibody responses (NAbs) to Clade C Tier 1A pseudovirus MW965.26 by four weeks post-vaccination with the CAP255 gp140 SpyTag SpyCatcher mi3 NPs, and two out of five developed neutralising activity to Clade C Tier 1B 6644 and Tier 2 ConC pseudoviruses (week 16, Figure 6C). However, none of the rabbits developed Tier 2 neutralising antibodies to the CAP255 pseudovirus. A second experiment was therefore carried out where two groups of rabbits were inoculated with two doses of the DNA vaccines mixed with CpG adjuvant (Env+Gag, 100 µg of each) at weeks 0 and 4, followed by three doses of nanoparticles at 8-week intervals (Figure 7A).

A group of five rabbits were inoculated with DNA vaccines expressing the CAP255 gp150 Env and mosaic Gag, followed by NPs decorated with the CAP255 gp140 SpyTag. All the rabbits developed potent NAbs responses to Tier 1A pseudovirus MW965.26 after the second NP inoculation, with ID50 values of up to 4851 (Figure 7B). Two out of the five rabbits developed NAb responses to the Tier 1B 6644 pseudovirus after the second NP inoculation, and this increased to three out of the five rabbits after the third NP inoculation (Figure 7B). Four out of the five rabbits in this group also developed weak NAb responses to the Tier 2 CAP256SU pseudovirus (Figure 7B). All five rabbits developed detectable, autologous Tier 2 neutralizing antibody responses to CAP255 (ranging from an ID50 value of 26 to 506; Figure 7B).

## 4. Discussion

In a previous study, we investigated displaying HIV-1 Env trimers on the surface of Acinetobacter phage AP205 VLPs, utilising the SpyTag/SpyCatcher system, as a potential HIV-1 vaccine [49]. However, the structural characterisation of these VLPs suggested that they were not uniformly saturated with Env trimers. It was concluded that the large size and symmetry of the Env trimer might not be ideally suited to display on an AP205 VLP, which is made up of 180 closely packed monomers. In this study, we investigated using the mi3 nanoparticle (60-mer) to display HIV-1 Env trimers.

A negative stain EM analysis showed that the SpyCatcher mi3 protein successfully assembled into uniform hollow icosahedral NPs of ~25 nm diameter (Figure 5A). SpyCatcher pentamers could be resolved at each of the 12 dodecahedral faces after image processing and 3D reconstruction. This density was clearly visible as low-resolution narrow disks in the centre of each dodecahedral face (Figure 5D,E) corresponding to the overall size and shape of five copies of the SpyCatcher protein (Figure 5F). This contrasts with the discrete SpyCatcher densities on the periphery of each of the twelve dodecahedral faces of SpyCatcher mi3 conjugated to the SpyTag SARS-CoV-2 spike protein receptor-binding domain (RBD) observed by Tan et al. (EMD-11997) [21]. The reason for this difference is not immediately apparent: in both cases, SpyCatcher is connected to the NP by a twelve amino acid linker, and there is almost complete sequence identity between the two mi3 NP constructs. We suspect that the differences are due to 22 additional N-terminal amino acids, the lack of a His-tag and the presence of a different conjugated antigen in the case of the Tan et al. NP. Interestingly, rings of density like ours were also observed in 3D classes of particles of mi3 linked via a flexible linker (GGS)4 to the humanFK506-binding protein imaged by cryo-EM [20].

After conjugation of the SpyCatcher mi3 NP with MBP SpyTag or CAP255 gp140 SpyTag, the NPs showed minimal distortion of the protein cage (Figure 5B,C). This observation is consistent with previous work [20,21] demonstrating the stability of the mi3 NP after conjugation or genetic fusion to binding partners. In raw images and after 2D classification, MBP SpyTag and CAP255 gp 140 SpyTag can be seen projecting outwards from the surface of the NP. Three-dimensional reconstruction of CAP255 gp140 SpyTag–SpyCatcher mi3 clearly shows that these additional densities originate at the centre of each of the dodecahedral faces of the NP (Figure 5G–I). This contradicted our original prediction, based on the EMD-11997 [21] structure, that 3-fold symmetric CAP255-gp 140 SpyTag would bind with 1:1 stoichiometry at the 3-fold icosahedral symmetry axes of SpyCatcher mi3. It explains our observation that increasing the ratio of CAP255 gp140 SpyTag to SpyCatcher mi3 in the coupling reaction did not lead to an increase in the coupled product or a decrease in unbound mi3 (Figure 4B) despite an excess of uncoupled Env SpyTag being present (Figure 4C).

The cryo-EM of CAP255 gp140 SpyTag–SpyCatcher mi3 and the 2D classification confirmed the assembly and stability of the mi3 NP core surrounded by regularly spaced CAP255 gp140 Env trimers (Figure 6A). Reconstructing the overall structure in 3D yielded a moderate average resolution of ~10 Å (Figure 6C) comprising a stable core NP surrounded by flexible Env trimers projecting from each of the pentagonal dodecahedral faces (Figure 6B,C). This flexibility is expected because, not only does the symmetry mismatch generate multiple possible conjugation patterns (Figure 6G,H), but CAP255 gp140, the SpyTag–SpyCatcher complex, and mi3 are all connected via flexible linkers (Figure 1A and Figure 4D). To better resolve the NP core, the CAP255 gp140 Env trimer SpyTag and SpyCatcher densities were masked during structural refinement, and icosahedral symmetry was applied, yielding a 5.4 Å resolution map (Figure 6D–F). The fold displays the expected α/β barrel structure and monomers superimposed onto the 2-keto-3-deoxy-phosphogluconate (KDPG) aldolase from *Thermotoga maritima* [52], computationally-designed NPs [53,54,55,56] and mi3 [21,22].

To better understand the structure of the overall complex, including the linker attachments, SpyCatcher protein and the average position of the conjugated CAP255 gp140 Env trimers, we reconstructed a single CAP255 gp140 Env trimer by masking out mi3 and 3D classifying the images without imposing symmetry. A regularisation parameter (T) = 20 led to a 17.7 Å resolution reconstruction (Figure 6D–F). The density corresponding to the shape of five copies of SpyCatcher could be seen in the 3D classified map. Despite not imposing symmetry, these formed a symmetrical arrangement that interacted across each of the twelve dodecahedral pentameric faces of mi3, forming 12 narrow disks (Figure 6F,G). The points of attachment of the linkers connecting SpyCatcher to mi3 are visible at the N-termini of mi3, as expected (Figure 6F,G). We used AlphaFold3 to model SpyCatcher mi3, which we fitted into the map using constrained flexible docking in Isolde (Croll, 2018). This allowed us to generate a pseudo-atomic model of the SpyCatcher mi3 NP for future use when predicting conjugation patterns.

To our knowledge, this is the first study to report the formation of SpyCatcher pentamers on the dodecahedral faces of mi3 NPs. More work will need to be undertaken to determine if this finding is generally applicable to the SpyCatcher mi3 system or is a unique feature of our construct. The resulting symmetry mismatch between trimeric Env SpyTag and pentameric SpyCatcher (Figure 6G–I) means that only twelve copies of Env, instead of the twenty predicted, are present on every NP. Despite this, Env is regularly displayed on the surface of the NP, in keeping with reports that the SpyCatcher mi3 system is resistant to symmetry mismatch [16]. Our work demonstrates the importance of fully characterising NPs by structural analysis and not relying on models to predict assembly properties, which affect the density and accessibility of epitopes.

Displaying stabilised HIV-1 Env trimers on nanoparticles (NPs) has been found to improve overall Env immunogenicity [25,57,58]. Repeating arrays of B- and T-cell epitopes on VLPs promote the T-cell independent cross-linking of B cells, as well as enhanced MHC presentation of T-cell epitopes, resulting in sustained and durable antibody and cellular responses, respectively [10,11,31]. In this study, rabbits vaccinated with the CAP255 Env developed autologous Tier 2 NAbs after the second nanoparticle immunisation and low levels of heterologous CAP256SU NAbs. The display of HIV Env on the surface of mi3 nanoparticles resulted in a more regular, repetitive display than when HIV Env was displayed on the surface of AP205 virus-like particles [49], which may have led to the improved antibody responses seen.

Sufficient yields of both the mi3 nanoparticles and HIV envelope protein can be produced for non-human primate studies using the methods described in this paper. Repeat harvests of Env from a single HYPERFlask^®^ (560 mL) yielded approximately 2.5 mg of protein. For larger quantities needed for clinical trials, Rahikainen et al. have developed a scalable, cost-effective method of preparing mi3 nanoparticles using ammonium sulphate precipitation followed by size exclusion chromatography [16]. Production and purification of the HIV envelope protein is more difficult, but various soluble HIV-1 envelope proteins have been produced for clinical trials. More recently, KBI Biopharma developed a platform strategy with which they were able to produce four gp120 envelope protein variants [59]. The Vaccine Research Centre at NIAID has developed a scalable manufacturing process using non-affinity resins for the purification of Env trimers [60], and the Duke Consortia for HIV AIDS Vaccine Development also have a GMP facility for the manufacture of HIV antigens.

The effect of Alhydrogel adjuvant on the stability of nanoparticles should be assessed in future experiments. Experiments have been carried out to assess the effect of Alhydrogel on the stability of BG505 and BG41 HIV envelope trimers [61]. Neither NS-EM nor blue native polyacrylamide gel electrophoresis could be used, as the alum interfered with these processes. However, differential scanning fluorimetry (DSF) could be used, and formulation of the trimers with Alhydrogel showed some stabilisation of the trimers. The authors also used biolayer interferometry (BLI) to assess the effect of Alhydrogel on the antigenicity of BG505 and BG41 by measuring the binding of bNAbs PGT145, PGT151 and 2G12 and non-Nab 19b. Similar experiments could be carried out with the CAP255-gp140 SpyTag–SpyCatcher mi3 NPs described in this paper.

One of the concerns with using nanoparticles as a vaccine platform is that high antibody responses are elicited to the nanoparticle itself, and it is possible these antibodies might competitively inhibit Env-specific responses or eliminate the NP itself after two or three immunisations [62]. The levels of mi3-specific antibodies were not measured in this study. However, the Tier 2 autologous NAbs were elicited after the second NP immunisation and boosted by the third immunisation. The second nanoparticle immunisation was a good indication that anti-mi3 responses after one immunisation did not prevent an immune response to the second nanoparticle immunisation. In other studies using ferritin [63] and I53-50 [24,25] NPs displaying HIV envelope proteins, NAbs were boosted with additional immunisations of NPs. In addition, presentation of the envelope proteins on NPs have consistently elicited better immune responses than soluble proteins [25,49,57,58].

A heterologous vaccination regimen was used in this study, as ours and other groups have shown that heterologous vaccination regimens are more effective at inducing higher magnitude and better quality HIV-specific immune responses than homologous regimens [49,51,64,65,66,67]. Inclusion of a modified vaccinia Ankara virus vaccine modality could further improve the levels of neutralising antibody titres. Ours and other groups have shown a DNA (D), MVA (M), protein (P) vaccination regimen (DDMMPP) elicits better neutralising antibody responses than other regimens, such as PPPP, MMPPP or DDMMM [34,68].

This study shows that HIV vaccines utilizing two-component nanoparticles, such as mi3 SpyCatcher and Env SpyTag, can be used to improve HIV-specific immune responses. Unlike in vivo self-assembling nanoparticles, two-component nanoparticles can be used to selectively display well-folded Env trimers because the antigens can be purified to high quality prior to coupling to the mi3 nanoparticle.

## Figures and Tables

**Table 1:** Live cell staining with MAbs of the HIV-1 envelope expressed on the surface of transfected cells.

Antibody	Neutralisation Breadth	Epitope	Native-Like Trimer	CAP255	CAP256SU
PG9	Broad	V2 apex	x	x	√
PGT128	Broad	V3 glycan supersite	x	√	√
VRC01	Broad	CD4 binding site	x	√	√
10E8	Broad	MPER	x	√	√
447-52D	Narrow *	V3 glycan	x	√	√
PGT145	Broad	V2 apex	√	x	√
VRC26.08	Broad	V2 apex	√	x	√
F105	Narrow	CD4 binding site	x	√	√

* Substantial cross-reactivity when V3 is exposed.

## Data Availability

The cryo-EM maps of unmasked CAP255 gp140 SpyTag–SpyCatcher mi3 and masked mi3 are deposited in the Electron Microscopy Data Bank under accession codes EMD-50635 and EMD-50615, respectively. The mi3 atomic model is deposited in the Protein Data Bank under ID 9fo3.

## Supplementary Materials

The following supporting information can be downloaded at: https://www.mdpi.com/article/10.3390/vaccines12091063/s1, Figure S1: Analysis of expression and Ni-NTA/IMAC purification of the His tagged SpyCatcher mi3.; Figure S2: Characterisation of the envelope on the surface of cells transfected with DNA vaccine expressing CAP255 gp150.; Table S1: EM data collection, refinement and validation statistics.
