# Supplementary material for: Development of a Two-Component Nanoparticle Vaccine Displaying an HIV-1 Envelope Glycoprotein that Elicits Tier 2 Neutralising Antibodies
Source: Vaccines (Basel). 2024 Sep 18;12(9):1063. doi: 10.3390/vaccines12091063 (PMC11436023; doi:10.3390/vaccines12091063)
Supplement: Supplementary file 1 [file vaccines-12-01063-s001.zip › vaccines-3132917-supplementary.pdf]

Table S1: EM data collection, refinement and validation statistics

**Data collection**	**NS-EM**		**Cryo-EM**		
Microscope	F20		Titan Krios		
Voltage (kV)	200		300		
Pixel size (Å)	3.12		0.831		
Defocus range (μm)	-1.5		-0.75 to -3.0		
Electron dose ($e^-/Å^2$)	50		20		
Number of frames per image	1		50		
Detector	DE16		K3		
**Map reconstruction**	**mi3**	**Env+mi3**	**mi3**	**Env+mi3**	**Env**
Number of images	89	50	27642	27642	27642
Initial particle number			500000	1439	1439
Final particle number			1439	1439	1439
Box size (pixels)	256	256	288	288	288
Symmetry	I	I	I	I	C1
Resolution (FSC = 0.143) (Å)	17	18	5.4	9.4	17.7
Map sharpening factor ($Å^2$)	NA	NA	-248	NA	NA
**Fit to map**					
Correlation coefficient (atoms)	0.97	0.76	0.92	0.79	0.87

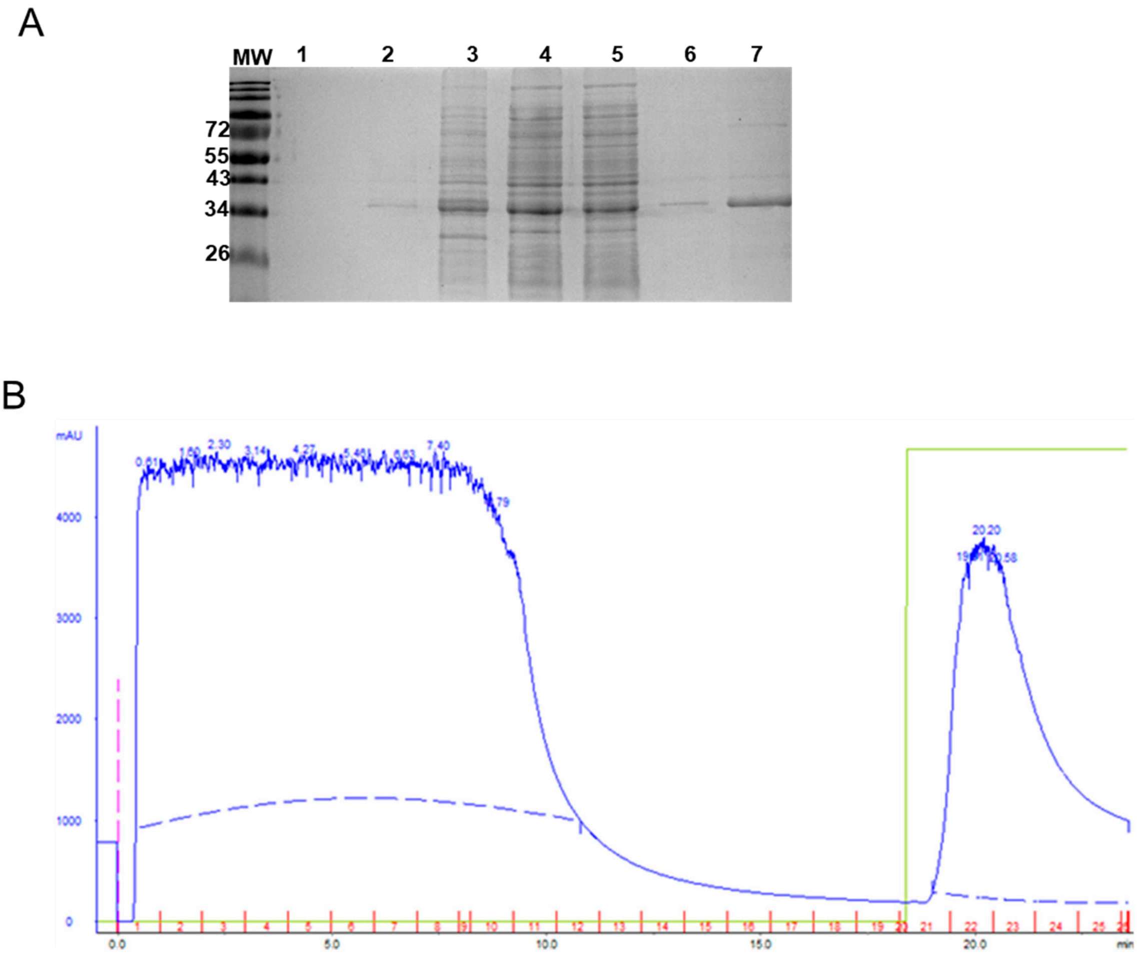

Figure S1. Analysis of expression and Ni-NTA/IMAC purification of the His tagged SpyCatcher-mi3. (A) 1- Uninduced culture, 2- IPTG Induced culture, 3- Pellet obtained following clarifying the lysate, 4- Total clarified lysate before purification, 5- Column flow through, 6- Wash flow through, 7- Eluate. MW- molecular weight (kDa). (B)The elution profile of the SpyCatcher-mi3 at UV 280 nm (blue line) in mAU. Time in minutes (black), fractions in red and the green line shows the one step elution.

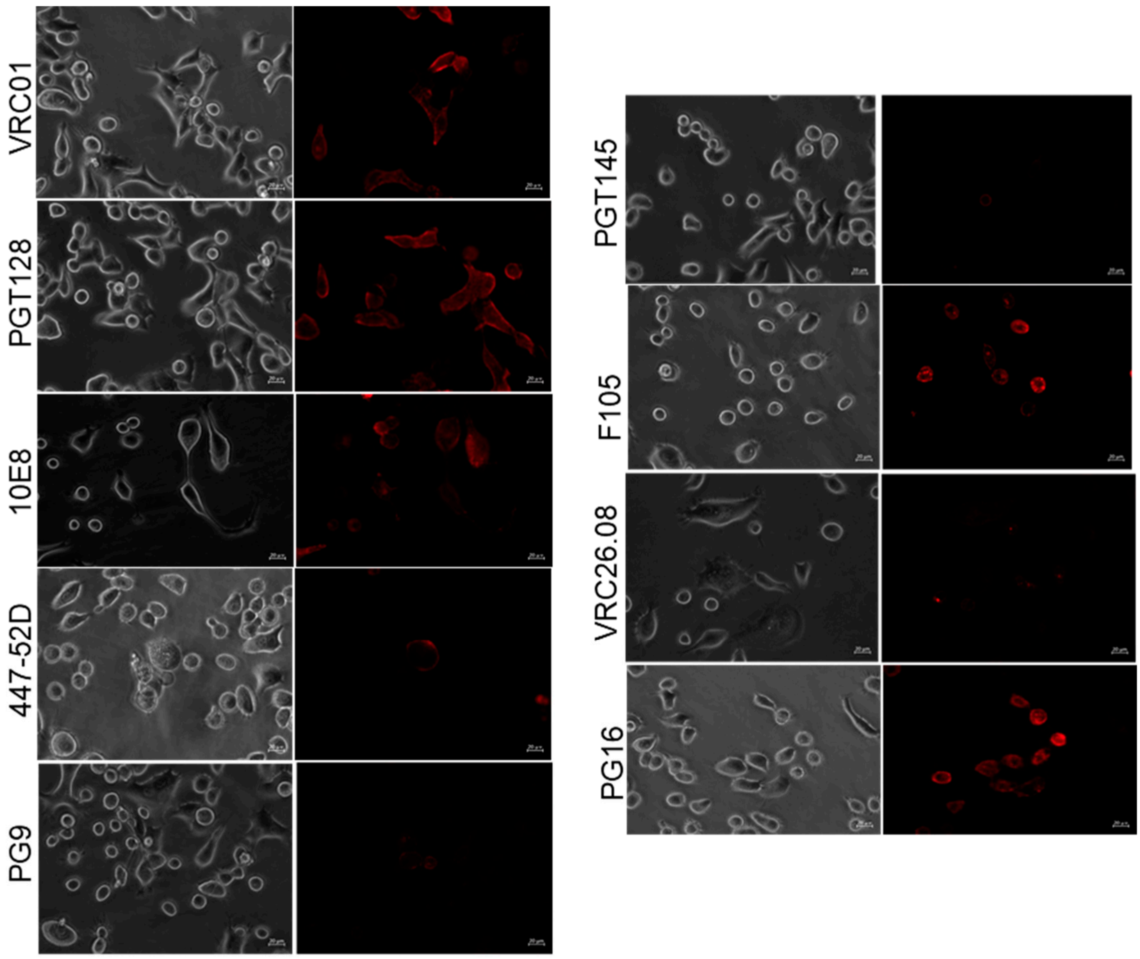

Figure S2. Characterisation of envelope on the surface of cells transfected with DNA vaccine expressing CAP255 gp150. HeLa cells transfected with pMExT-CAP255-gp150 and stained with MAbs VRC01, PGT128, 10E8, 447-52D, PG9, PGT145, F105, VRC26.08 and PG16. Bound MAbs were detected with anti-human IgG-Cy3 (red). TL = transmitted light, phase contrast.
